# Short-course pembrolizumab and continuous afatinib therapy for recurrent or metastatic head and neck squamous cell carcinoma: a real-world data analysis

**DOI:** 10.1186/s12885-022-10343-7

**Published:** 2022-11-28

**Authors:** Hsiang-Fong Kao, Huai-Cheng Huang, Bin-Chi Liao, Ruey-Long Hong

**Affiliations:** 1 Department of Medical Oncology, National Taiwan University Cancer Center, No.57, Ln. 155, Sec. 3, Keelung Rd, Da’an Dist Taipei, 106 Taiwan; 2 Department of Oncology, National Taiwan University Hospital, No.7, Chung Shan S. Rd, Zhongzheng Dist Taipei, 10002 Taiwan

**Keywords:** Head and neck squamous cell carcinoma, Pembrolizumab, Afatinib, Immunotherapy, Anti-PD-1

## Abstract

**Objectives:**

The optimal duration of anti-PD-1 for cancer therapy has not been tested, especially when using combination therapy. Epidermal growth factor receptor (EGFR) pathway blocker was the top compound that enhanced T-cell killing of tumor cells in a high-throughput immune-oncology screen, possibly by stimulate the antigen presentation machinery and other mechanisms. We explored the effect of combination of EGFR inhibition with a short course of anti-PD-1 therapy in patients with recurrent or metastatic head and neck squamous cell carcinoma (R/M HNSCC).

**Method:**

We analyzed the effect of a short course of anti-PD-1 with continuous afatinib on the survival of a real-world cohort of R/M HNSCC patients. Patient characteristics, treatments, efficacies, and toxicities were reviewed and recorded for analysis.

**Results:**

From November 2016 to May 2018, 51 consecutive patients received pembrolizumab and afatinib. The cutoff date was June 30, 2022. The most common toxicities (all grades) were diarrhea (62.7%), skin rash (43.1%), mucositis (31.4%), and paronychia (23.5%). The objective response rate was 54.9% (95% confidence interval [CI] 40.3–68.9%). Median progression-free survival was 5.9 months (95% CI: 4.4–7.6 months), and the median overall survival was 10.5 months (95% CI: 6.8–16.5 months). The 12-month, 24-month, 36-month, and 48-month survival rate was 47.0%, 22.5%, 17.7%, and 12.6% respectively.

**Conclusions:**

This retrospective study showed that short course pembrolizumab with afatinib therapy has acceptable efficacy in R/M HNSCC patients. The durable response and long-term survival rates were similar to prospective clinical trials. Short course anti-PD-1 therapy, especially in combination with EGFR blocker, is worth for further prospective study.

## Introduction

Anti-programmed death-1 (anti-PD-1) antibodies, including pembrolizumab [1, 2] and nivolumab [3], have been shown to be efficacious in the treatment of head and neck squamous cell carcinoma (HNSCC) in a first-line or platinum-refractory setting. However, the optimal duration of anti-PD-1 therapy for HNSCC patients has not been systemically investigated. In the CHECKMATE 141 study [3], the pivotal investigation of anti-PD-1 in HNSCC, enrolled patients took nivolumab until disease progression, intolerable toxicity, or patients' wishes to discontinue. In the KEYNOTE 040 [1] and KEYNOTE 048 [2] studies, patients received a maximum of 35 cycles (about two years) of pembrolizumab. For other types of cancer, the duration of application of anti-PD-1 or programmed cell death ligand-1 (PD-L1) antibody was different. The duration of treatment was 12 months in an adjuvant trial using durvalumab after concurrent chemoradiation in advanced non-small-cell lung cancer patients [4]. Ipilimumab, a cytotoxic T-lymphocyte-associated protein-4 (CTLA-4) antibody, showed a durable response in metastatic melanoma patients with only four cycles of therapy [5]. These studies showed the possibility of a shorter course of anti-PD-1 for cancer therapy.

Anti-PD-1 combination therapy has been used for overcoming intrinsic resistance and improving treatment efficacy in HNSCC. Several phase II studies have shown the effectiveness of epidermal growth factor receptor (EGFR) pathway inhibition for improving anti-PD-1 efficacy in HNSCC patients. Two studies that used cetuximab with pembrolizumab [6] or nivolumab [7] for HNSCC reported an improved response rate. A phase II study using afatinib also showed activity with pembrolizumab in platinum-refractory HNSCC patients [8]. The afatinib–pembrolizumab study also showed that afatinib may augment antigen-presentation machinery in the tumor microenvironment. Retaining afatinib in cancer therapy may be a strategy to maintain an inflamed microenvironment and to enhance the efficacy of anti-PD-1.

Combing with that best partner to enhance T-cell killing of tumor cells, a shorter course anti-PD-1 therapy may be feasible. We retrospectively reviewed real-world data from a medical center in Taiwan to explore the efficacy of a short course of pembrolizumab combined with continuous afatinib for HNSCC patients.

## Methods

### Ethics consideration

The medical record retrospective review focusing on recurrent or metastatic HNSCC patients taking anti-PD-1 was approved by the institutional review board (IRB) of the National Taiwan University Hospital (NTUH), Taipei, Taiwan (IRB approval number: 201710031RINB). Informed consent has been waived by the IRB of NTUH, Taipei, Taiwan, as this was a retrospective study. All methods were performed in accordance with the relevant guidelines and regulations.

### Data collection

Patients with head and neck cancer fulfilling the following criteria were enrolled for analysis: (1) documented HNSCC (excluding nasopharyngeal carcinoma); (2) recurrent or metastatic disease not suitable for further curative treatment; (3) no other concurrent cancers; and (4) taking a combination of pembrolizumab and afatinib for palliation. After obtaining positive results in a phase II trial [8], we focused on patients taking a short course of pembrolizumab with continuous afatinib in this real-world data set. Patients were excluded if they took pembrolizumab until disease progression or completing 35 cycles of therapy.

The study analyzed the following characteristics: age, the curative treatment plan, and history of chemotherapy or anti-cancer therapy. Patients' history of alcohol drinking, betel nut chewing, and cigarette smoking were also recorded. Definitive therapy included surgical tumor excision, radiotherapy alone, or chemoradiation. Time from definitive therapy to recurrence or metastases was defined from the last date of definitive therapy to the first date of known recurrence or metastases.

### Toxicity analysis

The medical records and laboratory data were reviewed for toxicity analysis. Severity was evaluated by certified medical oncologists and was recorded regularly in the electronic health record.

### Tumor response evaluation

Tumor response was evaluated using contrast imaging (magnetic resonance imaging or computed tomography). In the case of patients who did not have a contrast image evaluation, data on tumor size measured with a ruler were used in the analysis. RECIST 1.1 [9] was used to determine the clinical response of the patient.

### Survival analysis

We reviewed medical records as a reference for survival status. Progression-free survival (PFS) was defined as the period from the time of first taking pembrolizumab to the time of disease progression or death. Overall survival (OS) was defined as the period from the time of first taking pembrolizumab to the time of death. For responders defined by RECIST 1.1 [9], the duration of response (DoR) was the period from the time of the first image documenting partial response to the time of disease progression or death. The survival status of patients who did not have a regular follow-up was updated by cancer case managers in the cancer registry of the hospital. The last day of hospitalization of patients discharged from the hospital with critical illness was recorded as the date of death. If a patient's survival status could not be verified by medical records, the cancer registry, or telephone contact, the survival status was censored on the date of the last visit.

The Kaplan–Meier procedure was used for analysis of survival. The Cox proportional hazards regression model was used for multivariate analysis. The log-rank test was used to compare survival. MedCalc (Version 20.111) was used for the analyses.

## Results

### Patient characteristics

From Nov 2016 to Sep 2018, 51 consecutive HNSCC patients fulfilling the reviewing criteria were included in the analysis (Table 1). The day for data cutoff was June 30, 2022. Three patients were lost from follow-up before the data cutoff. The most common tumor site in the study was oral cancer (36/51, 70%). A review of treatment history showed that 90% (46/51) of the patients had prior radiotherapy, and 86% (44/51) were cetuximab-naïve. Within 180 days of definitive therapy, 26 patients (26/51, 51%) had a recurrence or distant metastases of the disease, and 19 of them were platinum-refractory.

**Table 1:** Patient characteristics

Characteristic	Afatinib-naïve
Patients, number	51
Gender, number (%)	
Male	48 (94%)
Female	3 (6%)
Age, median (range) (years)	59.2 (34.4–74.0)
Cancer type, number (%)	
Oral cavity	36 (70%)
Oropharynx	8 (16%)
p16 status: ( +) / (-) / NA	1 / 5 / 2
Hypopharynx	7 (14%)
Habits, number (%)	
Alcohol drinking	35 (69%)
Betel nut chewing	36 (71%)
Cigarette smoking	44 (86%)
Definitive treatment	
Surgery–CCRT	35 (69%)
Definitive CCRT	11 (21%)
Surgery alone	5 (10%)
Time from definitive therapy to recurrence/metastases (%)	
0–90 days	10 (20%)
91–180 days	16 (31%)
≥ 181 days	25 (49%)
Cetuximab, number (%)	
Naïve	44 (86%)
Induction	1 (2%)
Bio-RT	1 (2%)
For recurrent/metastatic disease	5 (10%)
Pembrolizumab, dose/cycle	
200 mg fixed dose	27 (52%)
2 mg/kg	25 (48%)
Pembrolizumab, cycles	
1–3 cycles	13 (25%)
4 cycles	34 (67%)
5–7 cycles	4 (8%)

The majority of patients (67%) took four cycles of pembrolizumab, and 52% of patients took a 200-mg fixed dose of pembrolizumab every three weeks. All patients took 40 mg of afatinib daily as the initial dose of treatment. The prescription of afatinib was maintained until disease progression, intolerable toxicity, or the physician's decision to discontinue. The median treatment duration for afatinib was 5.9 months (range: 0.5 to 25.8 months).

### Toxicities

For patients taking pembrolizumab and afatinib, the most common all-grade toxicities (Table 2) were diarrhea (62.7%), skin rash (43.1%), mucositis (31.4%), and paronychia (23.5%). The overall rate of grade 3–4 toxicities was 7.8% (4/51) (Table 2). Thirty-seven (72.5%) patients had dose reduction for toxicity. Tumor bleeding occurred in two patients after taking pembrolizumab and afatinib, and one patient’s death was caused by this condition. Both events were considered disease-related. One patient experienced grade 3 pneumonitis after pembrolizumab and afatinib treatment. Among the 51 patients in the cohort, 36 (70.6%) had dose reductions. After the dose was adjusted, the most common dose of afatinib was 40 mg daily in a 2 days on/1 day off schedule (32 patients).

**Table 2:** Treatment-related adverse events

Toxicity	All	%	Gr. 3–4	%
Diarrhea	32	62.7	1	2.0
Rash, skin	22	43.1	2	3.9
Mucositis	16	31.4	2	3.9
Paronychia	12	23.5	0	0.0
Weight loss	5	9.8	0	0.0
AST/ALT	4	7.8	0	0.0
Fatigue	4	7.8	0	0.0
Malaise	3	5.9	0	0.0
Fever	2	3.9	0	0.0
Edema, face	1	2.0	0	0.0
Nausea	1	2.0	0	0.0
Thrombocytopenia	1	2.0	0	0.0
Pneumonitis	1	2.0	1	2.0

### Clinical response and survival analysis

**Fig. 1:**
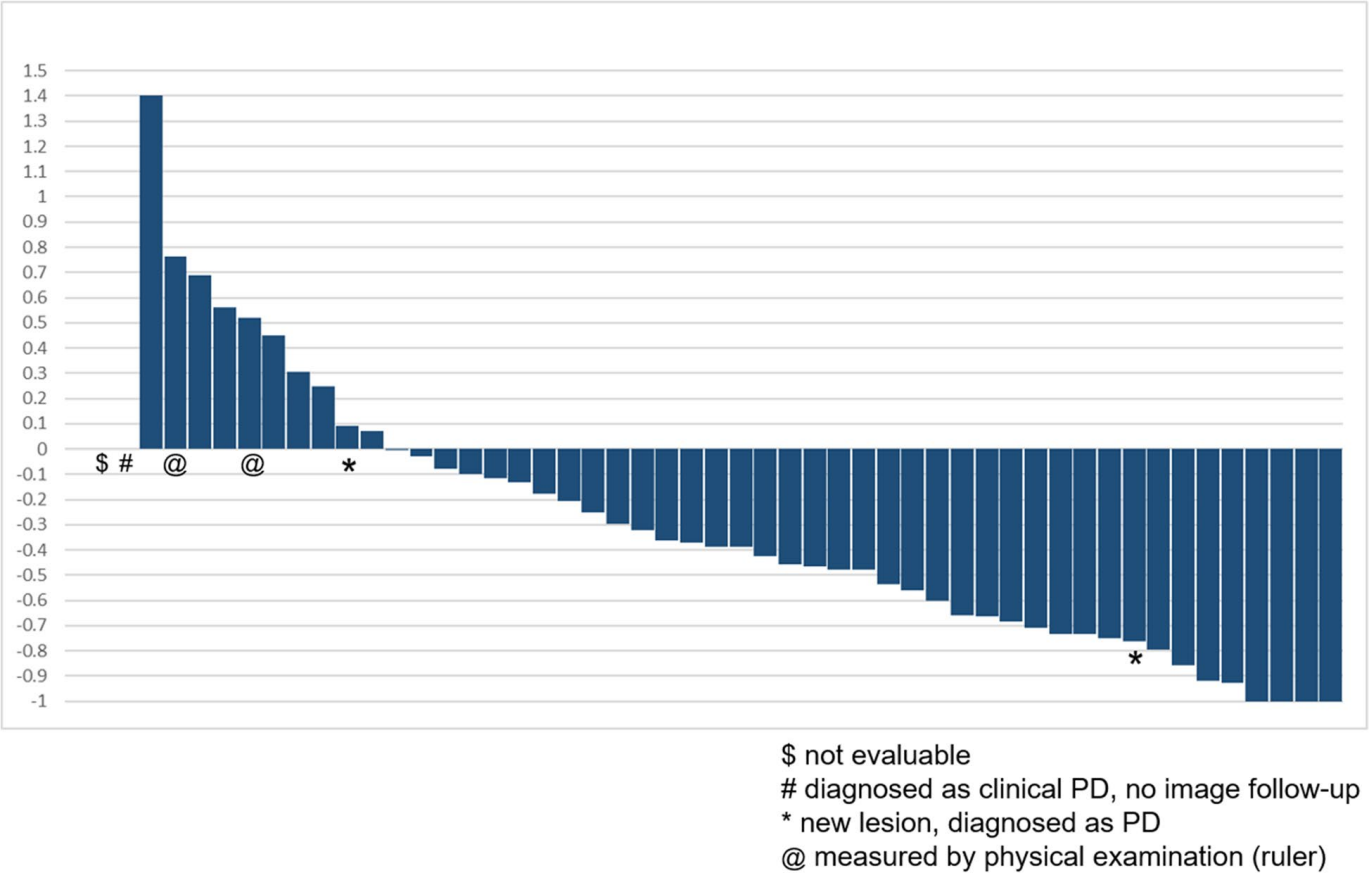
Clinical response in patients taking short-course pembrolizumab and continuous afatinib

In this cohort, 47 patients had contrast images for disease evaluation, while two patients were given a physical examination. The tumors were measured by rulers, and the size of tumor was recorded in the medical record. One patient was diagnosed with clinical progression. One patient did not have evaluable disease. In the case of the 47 patients with contrast images for evaluation, the median number of examinations of the contrast images was 3 (range: 2–12). The median interval of contrast image evaluation was 3.1 months (range: 0.5–20.4 months). The objective response rate (ORR) was 54.9% (95% CI, 40.3–68.9%) (Fig. 1), and all 28 responders had contrast images for evaluation. Eleven of the 51 patients (21.6%) had stable disease at first evaluation, and another 10 (19.6%) showed disease progression. The ORR of cetuximab-experienced patients was 57.1% (4/7).

**Fig. 2:**
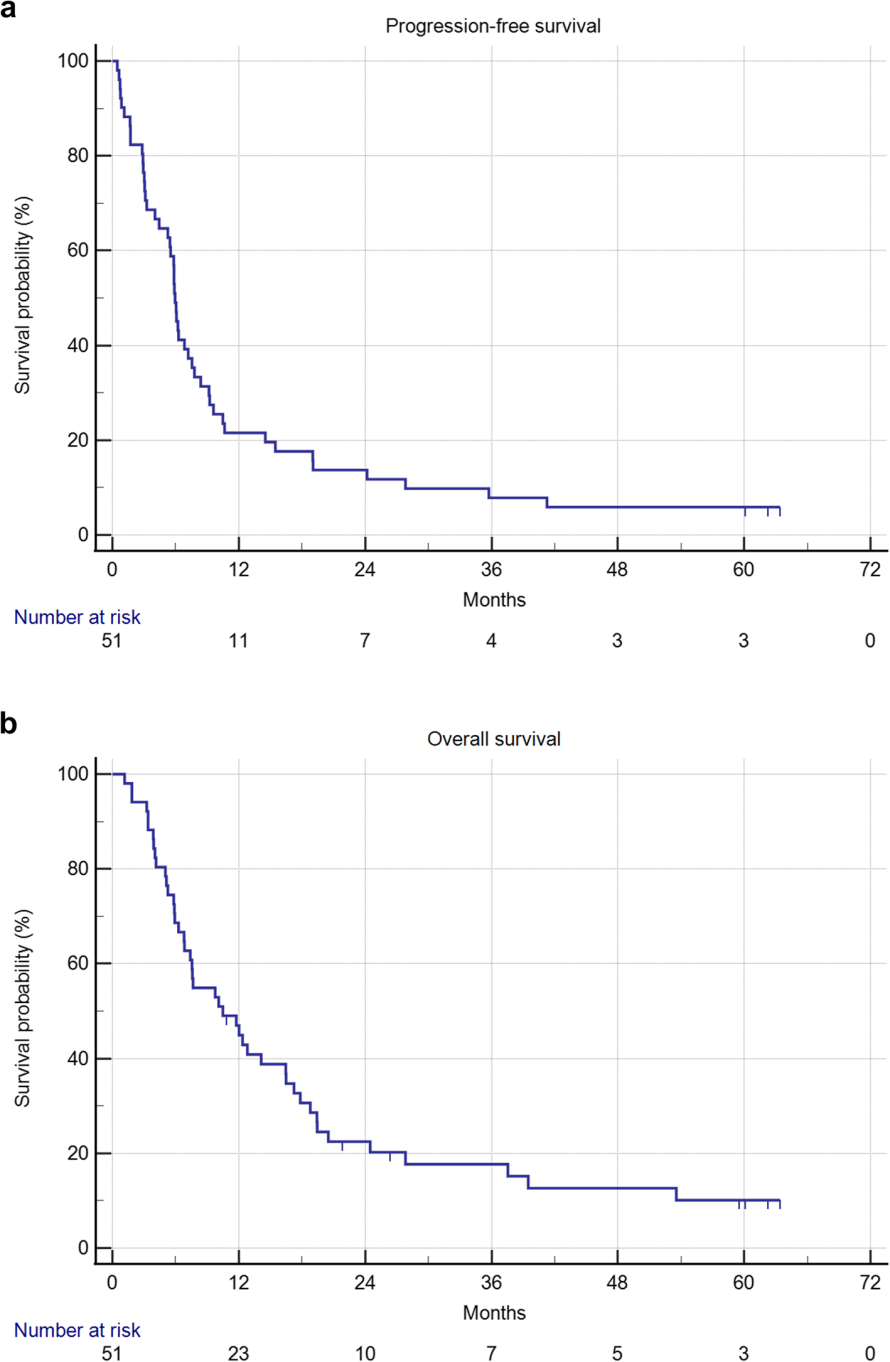
Survival analysis. 2A: Progression-free survival; and 2B: overall survival

The median PFS was 5.9 months (95% CI: 4.4–7.6 months; Fig. 2A), and the median OS was 10.5 months (95% CI: 6.8–16.5 month; Fig. 2B). The 12-month, 24-month, 36-month, and 48-month survival rate was 47.0%, 22.5%, 17.7%, and 12.6% respectively. The median duration of response of responders was 6.2 months (95% CI: 3.1–12.6 months).

Palliative therapies were continued in several patients following disease progression. Patients received chemotherapy in 37 cases, cetuximab in 25 cases, anti-PD-1 in 9 cases, and ipilimumab in 2 cases. A total of eight patients received radiotherapy for palliation. In nine cases, no further therapy was continued.

### Subgroup analysis

**Fig. 3:**
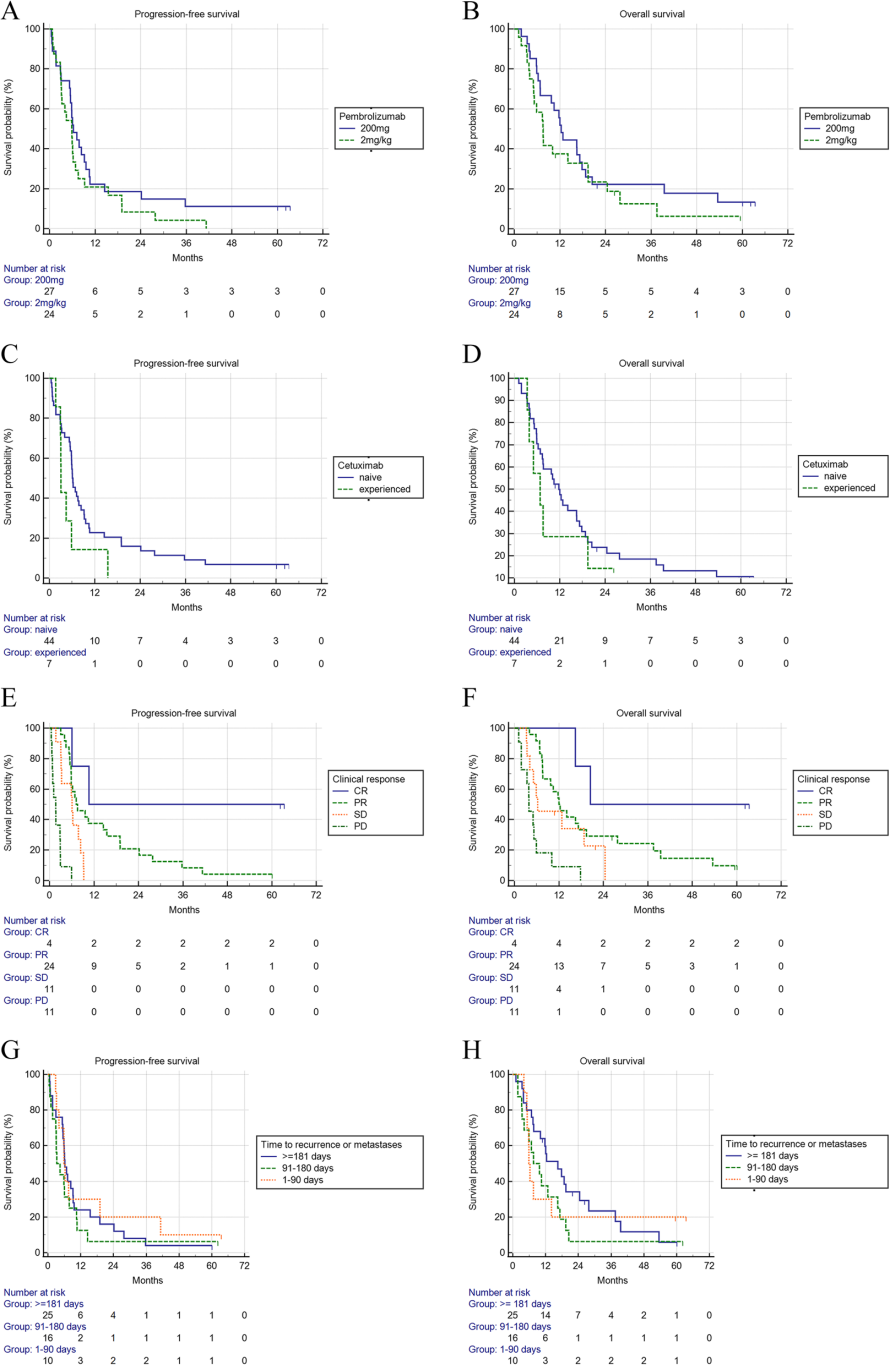
Subgroup survival analysis. Survival analysis under different pembrolizumab dose – 3A: PFS; 3B: OS. Survival analysis under cetuximab use – 3C: PFS; 3D: OS. Survival analysis under different clinical response states – 3E: PFS; 3F: OS. Survival analysis under different status of recurrence – 3G: PFS; 3H: OS

We included the history of alcohol drinking, cigarette smoking, betel nut chewing, cetuximab use, cancer types (oral vs. non-oral), time from definitive treatment to recurrence, and pembrolizumab dose (200 mg vs. 2 mg/kg) in the Cox proportional hazards regression analysis of OS. No difference was observed in this analysis (Table 3). There was no significant difference in PFS (log-rank *p* = 0.23, Fig. 3A) and OS (log-rank *p* = 0.31, Fig. 3B) between pembrolizumab doses. Regarding the exposure of cetuximab, there was no significant difference in PFS (log-rank *p* = 0.07, Fig. 3C) and OS (log-rank p = 0.43, Fig. 3D). In these cohorts, the response to the therapy predicted the PFS and OS benefits (log-rank test of PFS: *p* < 0.0001; Fig. 3E; log-rank test of OS: *p* < 0.0001; Fig. 3F). Regarding different time from definitive treatment to recurrence or metastases, there was no difference in PFS (Fig. 3G, log-rank *p* = 0.47) and OS (Fig. 3H, log-rank *p* = 0.35).

**Table 3:** Cox proportional-hazards regression analysis

	HR (95% CI)	*p*-value
Time from definitive therapy to recurrence (≤ 180 days vs. > 180 days)	1.42 (0.69, 2.92)	0.348
Cancer type (oral vs. non-oral)	0.46 (0.19, 1.14)	0.095
Alcohol drinking (no vs. yes)	0.88 (0.38, 2.01)	0.753
Betel nut chewing (no vs. yes)	1.89 (0.74, 4.85)	0.186
Cigarette smoking (no vs. yes)	0.44 (0.14, 1.33)	0.145
Cetuximab (experienced vs. naïve)	2.32 (0.82, 6.54)	0.111
Pembrolizumab dose ( 2 mg/kg vs 200 mg fixed dose)	1.71 (0.82, 3.60)	0.154

## Discussion

This is a retrospective analysis of real-world data for patients using limited cycles of pembrolizumab with continuous afatinib for the treatment of HNSCC. The median cycles of pembrolizumab treatment are four cycles, approximately before the first image evaluation. It was an appropriate time for discussing further treatment plans with the patients and their family. In this retrospective analysis, the reasons for holding pembrolizumab treatment are not written down in medical records. However, financial restraint could be the most possible reason to hold the therapy. In this study, the treatment of short-course pembrolizumab plus continuous afatinib still showed a comparative clinical effect. The ORR of recurrent and/or metastatic HNSCC patients to the treatment was 54.9%. The median PFS was 5.9 months, and the median OS was 10.5 months. The toxicities were tolerable and manageable. The response rate was comparable to that in the prospective phase II study using afatinib–pembrolizumab for HNSCC [8]. The long-term survival rate of this study were also close to the results of CHECKMATE 141 [10], KEYNOTE 040 [1, 11], and KEYNOTE 048 [2]. The 24-month survival rate of 22.5% was numerically similar to the long-term follow-up results in CHECKMATE 141, which showed a 24-month survival rate of 16.9% in the nivolumab arm [10]. The 48-month survival rate of this study was 12.6%, which was also similar to the result of KEYNOTE 040, with a 48-month survival rate of 8.3% in pembrolizumab arm [1]. These indirect comparisons demonstrate the feasibility of short-course pembrolizumab with continuous afatinib therapy for HNSCC patients.

In addition to regulating tumor growth [12], the EGFR pathway also regulates tumor microenvironment [13, 17]. EGFR inhibition can increase MHC expression, enhance dendritic cell function, and increase T cell infiltration into the tumor [13]. In a cancer animal model, afatinib could suppress tumorigenesis by inhibiting the EGFR pathway of macrophages [14]. In a syngeneic mouse cancer model, EGFR TKI suppressed the glycosylation of PD-L1 and sensitized the tumor to anti-PD-1 therapy [15]. In a drug screening study using ovalbumin-specific systems, EGFR TKI, especially afatinib, could enhance the efficacy of anti-PD-1 by improving MHC expression and decreasing PD-L1 expression in tumor cells [16]. These studies support EGFR-targeted therapy, especially afatinib, as a potent partner for checkpoint inhibitor immunotherapy [17]. A phase II study using afatinib and pembrolizumab showed an improved ORR in HNSCC patients [8]. The post-treatment multi-omic analysis in the study also showed enhanced antigen presentation machinery in the tumor microenvironement after taking afatinib and pembrolizumab [8]. These studies indicate the potential of EGFR inhibition for augmenting the efficacy of anti-PD-1 in HNSCC patients.

The high costs of anti-PD-1 and reimbursement constraints limit patients' access to anti-PD-1 therapy [18]. In Taiwan, anti-PD-1 therapy was not reimbursed by National Health Insurance until 2019 [19]. Therefore, a shorter-course and lower-dose anti-PD-1 treatments is highly attractive to patients with financial constraints. The Cox proportional hazards regression analysis in our study showed that a lower dose of pembrolizumab (2 mg/kg) may have OS benefits similar to those of the standard dose (Table 3, Fig. 3A, 3B). The analysis demonstrated the potential of low-dose short-course pembrolizumab therapy for treating HNSCC patients. In KEYNOTE 010, a phase III study used two doses of pembrolizumab (2 mg/kg and 10 mg/kg), which also resulted in similar survival benefits [20]. In a real-world study, a lower dose of anti-PD-1 did not result in inferior survival benefits to non-small-cell lung cancer patients [21]. This evidence shows the feasibility of lower-dose anti-PD-1 as cancer therapy.

The duration of anti-PD-1 treatment is also of interest. In metastatic melanoma patients, four cycles of the CTLA-4 antibody ipilimumab demonstrated a durable response [5]. The survival benefits are not compromised for patients who discontinue anti-PD-1 therapy due to immune-related adverse events [22, 23]. Observations from these clinical studies indicate that a shorter course of anti-PD-1 could be an effective alternative to chemotherapy. Our study also showed that patients with clinical response (CR or PR) have a better prognosis than patients with stable disease or disease progression (Fig. 3E, 3F). Therefore, patients with a good clinical response may be suitable for a shorter course strategy. A prospective study is ongoing to ascertain whether short-course anti-PD-1 is feasible for melanoma patients with clinical response [24]. The study may show the potential utility of a short course of anti-PD-1 in cancer therapy.

The report also identified several challenges that require further investigation. A key issue in TKI and anti-PD-1 combination therapy is the management of TKI associated toxicities. In this cohort, 70.6% of patients required dose reductions. In other clinical trials using afatinib-pembrolizumab [8], lenvatinib-pembrolizumab [25] or carbozantinib-pembrolizumab [26] combination therapies in HNSCC, a significant portions of patients required dose adjustment. A appropriate dose selection or a different dosing schedule for TKIs is essential for further utilizing anti-PD1 and TKI combinations. In addition, the median DoR of this cohort was 6.2 months, which is shorter than the median DoR of pembrolizumab monotherapy in KEYNOTE 040 [1] and KEYNOTE 048 [2] trials. It is possible that afatinib may not effectively modulate the microenvironment in every responders in this cohort. The key to extending the duration of response of anti-PD-1 combinations is to study the difference of microenvironment between each durable responder and non-durable responder.

This study has several limitations. First, it is a retrospective study. Efficacy might have been overestimated and toxicities underestimated. The heterogeneous characteristics in our study also limited further subgroup analysis for predicting treatment benefits. Second, the cost of these treatment was not reimbursed in Taiwan in this retrospective study period. Patients who can afford the cost of therapy may reflect a better family support and financial status. The prognosis of these patients could be better in this cohort. Lastly, PD-L1 expression serves as a biomarker for predicting clinical response to anti-PD-1 therapy. However, PD-L1 testing is not widely available in Taiwan at this time. As a result, it may be difficult to determine the correlation between PD-L1 expression and clinical response rate. Our prospective study found that higher PD-L1 expression was associated with better outcomes in patients with HNSCC receiving afatinib-pembrolizumab [8].

In conclusion, this retrospective analysis of real-world data showed acceptable efficacy of short-course pembrolizumab with continuous afatinib against HNSCC. The substantial long-term survival rates support the possibility of using short-course pembrolizumab for HNSCC. Short course of pembrolizumab also provides incentives to reduce medical costs. The feasibility of short-course anti-PD-1 warrants a prospective randomized study to confirm its efficacy.

## Data Availability

The datasets used and/or analysed during the current study available from the corresponding author on reasonable request.
